# Delayed diagnosis of a cesarean scar pregnancy: a case report

**DOI:** 10.1186/s13256-019-1983-1

**Published:** 2019-03-07

**Authors:** Eun Ju Jo, Hyun-Hwa Cha, Won Joon Seong

**Affiliations:** Department of Obstetrics and Gynecology, Kyungpook National University Hospital, School of Medicine, 807 Hogukro, Buk-gu, Daegu, 41404 Republic of Korea

**Keywords:** Ectopic pregnancy, Cesarean scar, Laparotomy, Delayed diagnosis

## Abstract

**Background:**

Cesarean scar pregnancy is rare but may be related to early uterine rupture and may result in massive hemorrhage. Nowadays, most cesarean scar pregnancies are diagnosed early and can be managed properly. However, diagnoses of cesarean scar pregnancies that develop in the obstetrical area are sometimes delayed.

**Case presentation:**

A 28-year-old Asian woman visited our institution because of suspected cesarean scar pregnancy. Ultrasonography and computed tomography confirmed a cesarean scar pregnancy with a live fetus with a crown-rump length of 4.83 cm, corresponding to 11 weeks 6 days of gestation. Initially, we injected 50 mg of methotrexate in the amniotic sac under transabdominal ultrasonographic guidance. However, fetal cardiac activity was still observed 2 days later. We decided to perform open laparotomy because of the possibility of massive bleeding. The gestational sac was removed, as well as most of the trophoblastic tissues that were adherent and invading the wall of the lower uterine segment. She was discharged in good condition 5 days after the operation.

**Conclusions:**

Despite the popular use of ultrasonography in prenatal care, diagnosis of cesarean scar pregnancy is still delayed. Surgical treatment with local methotrexate injection could be an option for the management of advanced cesarean scar pregnancy.

## Background

Cesarean scar pregnancy (CSP) is the rarest form of ectopic pregnancy and results in implantation of the gestational sac into the fibrous tissue scar of a previous cesarean section. CSP accounts for 6.1% of ectopic pregnancies, and 0.15% of pregnancies in which the patient had previously undergone a cesarean section [1, 2]. It is considered potentially lethal, as it leads to high risks of uterine rupture. Therefore, early diagnosis and prompt treatment are important to reduce maternal morbidity and mortality. Fortunately, appropriate prenatal care using ultrasonography (USG) permits earlier and accurate diagnosis of CSP; therefore, few cases of CSP diagnosed at a gestational age > 10 weeks have been reported. Here, we report a case of delayed diagnosis of CSP at 11 weeks and 6 days of gestation, for which open laparotomy was performed.

## Case presentation

A 28-year-old Asian woman (G3P1) who had undergone emergency cesarean delivery owing to a compound presentation at full term was referred to our institution with a suspicion of abnormally located gestational sac. She had undergone laparoscopic cholecystectomy and open appendectomy previously. She did not have any medical, family, or psychosocial history. She had missed her menstrual period without any other symptom and visited a private obstetrical clinic to confirm the pregnancy. However, she was diagnosed as having an abnormal pregnancy such as cervical or CSP by USG.

**Fig. 1:**
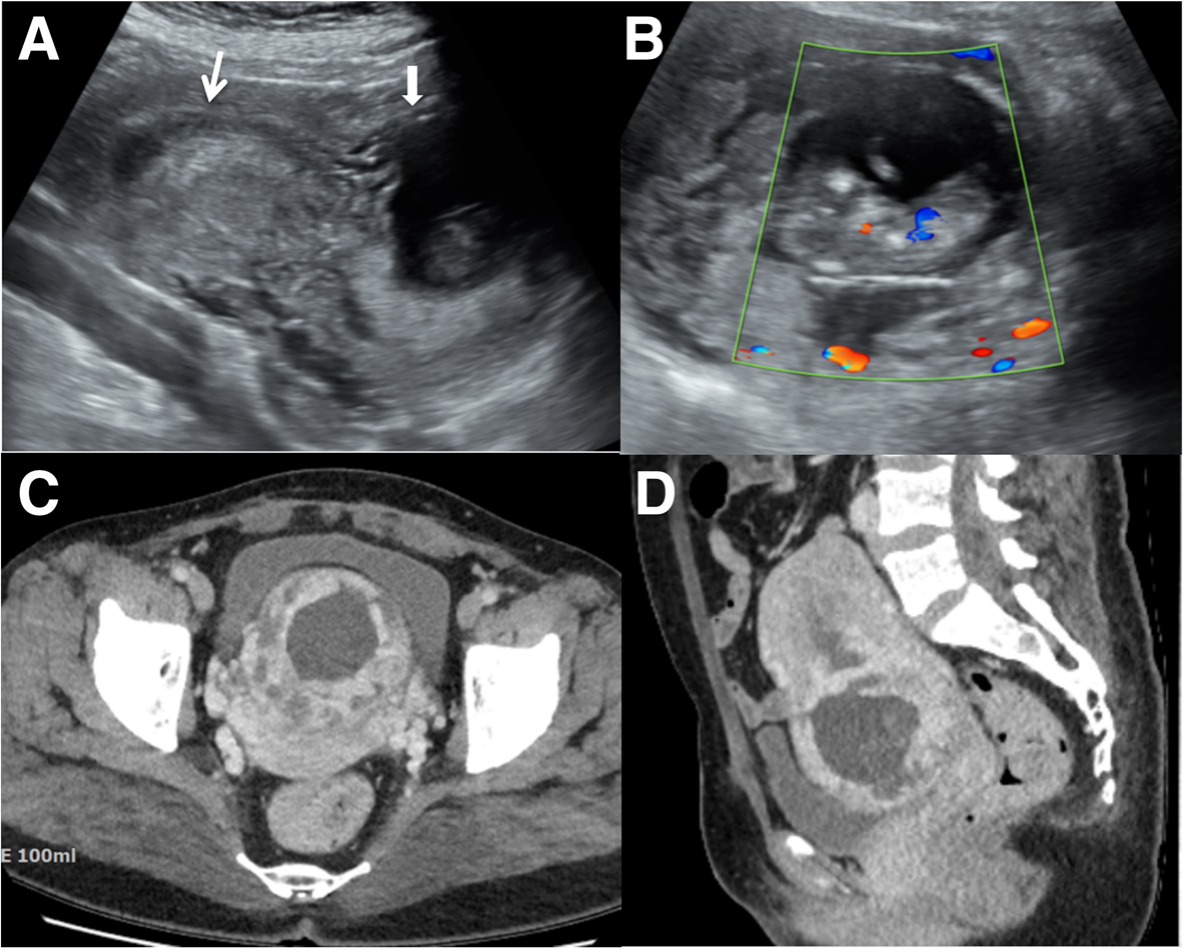
The initial transabdominal ultrasonography images show a gestational sac (bold arrow) located anteriorly in the lower uterine cavity with an empty uterine endometrial cavity (narrow arrow) (**a**). The color/power Doppler images depict a hyperechoic rim of choriodecidual reaction with excessive vascularity (**b**). The computed tomography image shows an intrauterine gestational sac in the lower uterine segment bulging through the anterior uterine wall at the site of the cesarean scar without invasion of the urinary bladder (**c, d**)

**Fig. 2:**
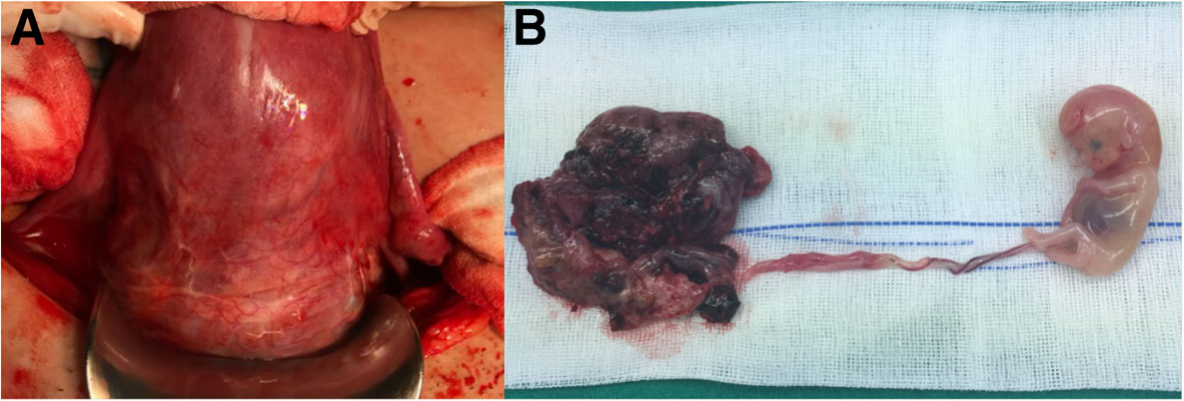
Bulging lower segment of the uterus observed during open laparotomy (**a**). The gross finding shows the placenta and fetus measuring 6.0 × 2.4 cm without other gross anomalies (**b**)

At our institution, she reported that her last menstrual period was just 5 to 6 weeks prior. However, USG revealed a gestational sac in the anterior lower uterine segment with a fetus measuring 4.83 cm crown-rump length (CRL) with positive cardiac activity, corresponding to 11 weeks and 6 days of gestation. Color/power Doppler images depicted a hyperechoic rim of a choriodecidual reaction with excessive vascularity (Fig. 1). Although we could observe a definitive abnormally located gestational sac, our patient did not have any pain during the physical examination. She admitted that her last menstrual period was different from her usual menstrual periods. Because CSP or cervical pregnancy was suspected, we performed computed tomography (CT) for a definitive diagnosis. The CT scan showed an intrauterine gestational sac in the lower uterine segment bulging through the anterior uterine wall at the site of the cesarean scar. No invasion of the urinary bladder was observed (Fig. 1). On presentation, her β-human chorionic gonadotropin (β-hCG) level was 66,536.8 IU/L (Day 1). Initially, we injected 50 mg of methotrexate (MTX) mixed with 9 mL of normal saline in the amniotic sac through a 22-G needle transabdominally under USG guidance. Simultaneously, 2 ml of amniotic fluid was aspirated for termination of the pregnancy. However, fetal cardiac activity was still observed 2 days later (Day 3), without significant changes in the serum β-hCG levels (65,342.5 IU/L). We decided on laparotomy instead of laparoscopy because of the large CRL (Day 4). The intraoperative finding showed bloody amniotic fluid, blood clot, placenta, and a fetus at the lower segment of the uterus. A transverse uterine incision was made at the lower segment of the uterus (Fig. 2). The gestational sac was removed, as well as most of the trophoblastic tissues that were adherent and invading the wall of the lower uterine segment. The fetus and placenta showed no definitive abnormalities (Fig. 2). The estimated blood loss was 1.2 L at intra-operation, without immediate complication. The uterine defect was repaired into two layers by using 2–0 Vicryl sutures. Our patient received 3 units of packed red blood cells (PRBC) at the ward postoperatively. The serial β-hCG level was 1958 IU/L at 4 days after the surgery (Day 8). She was discharged in good condition 5 days after the operation (Day 9). After 1 month (Day 39), her β-hCG levels returned to normal (2.8 IU/L). She was very satisfied with the fact that she had recovered well without the need for intensive care or further treatment without the need for hysterectomy.

## Discussion

CSP is a rare form of ectopic pregnancy. Its exact pathogenesis is unknown, but attributing factors have been thought to include endometrial and myometrial disruption or defects, or microscopic isolation between the cesarean scar and the space of the endometrium and implantation of the conceptus in the myometrium through the tract by the invading blastocyst [3, 4]. Based on imaging findings and progress reports during pregnancy, CSP is divided into two types: type 1 and 2. In type 1 CSP (endogenic type), implantation occurs on the scar site and the gestational sac grows toward the cervico-isthmic or uterine cavity. Type 2 CSP (exogenic CSP) occurs when the gestational sac is deeply embedded in the scar and the surrounding myometrium, and grows toward the urinary bladder. Although the exogenic type of CSP carries a greater risk of earlier uterine rupture, several cases of viable birth following the diagnosis of the endogenic type of CSP have been reported [5, 6]. However, expectant management of CSP that leads to live birth at the late preterm period is known to be associated with severe maternal morbidity such as massive hemorrhage, uterine rupture, or hysterectomy [7]. Our case was an endogenic type of CSP; therefore, it could continue until 12 weeks of gestation without uterine rupture. However, we decided to terminate the pregnancy owing to the consideration of maternal morbidity.

Earlier detection and appropriate management of CSP are important. Transvaginal USG is extremely helpful in the diagnosis of CSP with the following criteria: an empty endometrial cavity and cervical canal, a gestational sac in the anterior uterine wall, and prominent trophoblastic/placental blood flow [1–3]. Magnetic resonance imaging (MRI) is superior in the assessment of pelvic structures because of improved differentiation of soft tissue, spatial resolution, and the possibility of multiplanar imaging. Sagittal and transverse T1-weighted and T2-weighted MRI sequences can clearly show the gestational sac embedded in the anterior lower uterus. Furthermore, the T2-weighted sagittal section is the best view for the cesarean section scar, gestational sac, and decidual layer of a CSP [8]. However, MRI takes a long time to perform and does not require serial follow-up; therefore, many clinicians prefer USG. Despite these diagnostic tools, diagnosis of CSP is still delayed. In this case, she had mistaken abnormal vaginal bleeding for normal menstrual cycle. Therefore, the diagnosis was delayed until 12 weeks of gestation.

Although the incidence of CSP has increased in recent decades, a definitive treatment modality has not been established yet [4]. The treatment of CSP should be individualized on the basis of the clinical presentation, gestational age, type of pregnancy on imaging, and fertility preservation. Medical treatment using MTX is usually attempted systemically or locally before surgical treatment [7, 9, 10]. Previous studies have reported that an earlier gestational age and faster diagnosis result in a higher success rate of conservative treatments with MTX [5, 11, 12]. Local MTX therapy can increase the concentration of MTX in one area and reduce side effects compared to systemic therapy [13]. Systemic MTX therapy seems to be relatively effective in patients with β-hCG levels of < 5000 IU/L [14]. Surgical treatment is not recommended except in cases where the conservative treatment fails due to high morbidity and poor prognosis or in cases of uterine rupture due to late diagnosis [15]. Dilation and curettage, vacuum extraction method, hysteroscopy, laparoscopy, and laparotomy can be operative treatment options [16–19]. Recent reports have suggested that hysteroscopy offers little benefit when compared with curettage alone [20]. Laparoscopic procedures should be performed by a highly skilled surgeon, and vasopressin injection or laparoscopic coagulation or ligation of the uterine artery may be used to reduce bleeding [21]. Interventional radiological techniques such as uterine artery embolization have been successfully used. One of the serious complications of uterine artery embolization is pulmonary embolism. As pulmonary embolism is rare but life-threatening, obstetricians should be aware of the potential for serious complications after uterine artery embolization procedures and should evaluate the risk of embolization as a treatment option [22]. Also, because our patient has only one living child, we chose laparotomy instead of uterine artery embolization in this case.

In this case, the diagnosis of CSP was delayed and thus the risk of bleeding was higher. There was no established definition of advanced or delayed diagnosed CSP. However, in most previous reports, CSP was diagnosed between 6 and 8 weeks of gestation [2, 3, 5, 8]. Therefore, we had difficulty in choosing the treatment option. To reduce the possibility of maternal morbidity, we locally injected MTX with aspiration of amniotic fluid. We assumed that the procedure would help reduce the risk of bleeding.

## Conclusion

Although the diagnosis of CSP is rarely delayed, its treatment is important because it can be life-threatening. However, no treatment for CSP has been established and few cases have been reported. More cases and multicenter cohort studies are needed, and laparotomy with local MTX injection may be a treatment option.

## Abbreviations

CRL: Crown-rump length
CSP: Cesarean scar pregnancy
CT: Computed tomography
MRI: Magnetic resonance imaging
MTX: Methotrexate
PRBC: Packed red blood cells
USG: Ultrasonography
β-hCG: β-human chorionic gonadotropin
